# Adolescent Self-control Predicts Midlife Hallucinatory Experiences: 40-Year Follow-up of a National Birth Cohort

**DOI:** 10.1093/schbul/sbu050

**Published:** 2014-04-08

**Authors:** Atsushi Nishida, Kate Man Xu, Tim Croudace, Peter B. Jones, Jenifer Barnett, Marcus Richards

**Affiliations:** ^1^Department of Psychiatry & Behavioral Science, Tokyo Metropolitan Institute of Medical Science, Tokyo, Japan;; ^2^MRC Unit for Lifelong Health and Ageing at UCL, London, UK;; ^3^Department of Psychiatry, University of Cambridge, Cambridge, UK;; ^4^Department of Health Sciences and Hull York Medical School, University of York, York, UK;; ^5^CAMEO, Cambridgeshire & Peterborough NHS Foundation Trust, Cambridge, UK;; ^6^Cambridge Cognition Ltd, Cambridge, UK

**Keywords:** self-control, adolescent, psychotic-like experiences, longitudinal, conduct problems

## Abstract

*Background*: Associations between self-control in adolescence and adult mental health are unclear in the general population; to our knowledge, no study has investigated self-control in relation to psychotic-like symptoms. *Aims*: To investigate the relationship between adolescent self-control and the midlife mental health outcomes of anxiety and depression symptoms and psychotic-like experiences (PLEs), controlling for the effect of adolescent conduct and emotional problems and for parental occupational social class and childhood cognition. *Methods:* A population-based sample, the MRC National Survey of Health and Development (the British 1946 birth cohort) was contacted 23 times between ages 6 weeks and 53 years. Teachers completed rating scales to assess emotional adjustment and behaviors, from which factors measuring self-control, behavioral, and emotional problems were extracted. At age 53 years, PLEs were self-reported by 2918 participants using 4 items from the Psychosis Screening Questionnaire; symptoms of anxiety and depression were assessed using the scaled version of the General Health Questionnaire (GHQ-28). *Results:* After adjustment for the above covariates, poor adolescent self-control was associated with the presence of PLEs in adulthood, specifically hallucinatory experiences at age 53 years, even after adjustment for GHQ-28 scores. *Conclusions:* Lower self-control in adolescence is a risk factor for hallucinatory experiences in adulthood.

## Introduction

Self-control is defined as “the capacity to override natural and automatic tendencies, desires, or behaviors”; “to pursue long-term goals, even at the expense of short-term attractions”; and “to follow socially prescribed norms and rules.” In other words, self-control is the capacity to alter the self’s responses to achieve a desired state or outcome that otherwise would not arise naturally.^1^ Self-control pertains to the ability to wilfully or voluntarily inhibit, activate, or modulate attention and behaviors, as well as executive functioning tasks of planning, detecting errors, and integrating information relevant to selecting behavior.^2^

Recent reports from birth cohort studies have indicated that self-control in childhood and adolescence might be a significant developmental precursor that could predict a broad range of later outcomes including educational and occupational underachievement, poor physical health, and trouble with the criminal justice system.^3,4^ However, these studies did not identify clear associations between self-control and mental health outcomes. This is an important issue given the impact of these outcomes. For example, common mental disorders are a leading cause of years of health lost to disease in middle- and high-income countries, and its economic costs can be huge.^5^ Based on the longitudinal data of the Dunedin Multidisciplinary Health and Developmental Study, Moffitt et al^3^ found a significant inverse association between self-control in childhood and adolescence and substance dependence but found no such association with recurrent depression at age 32 years. In contrast, Fergusson et al^4^ found that self-control in childhood and adolescence was associated with later anxiety disorder and suicidal ideation (although not major depressive disorder) at age 30 years after controlling for sex, IQ, and socioeconomic status. However, these associations were explained by childhood conduct problems, which are well known to predict adverse life outcomes, including mental health problems.^6–8^ On the other hand, studies have reported continuity between emotional problems in adolescence and later depression and other mental health outcomes,^9–13^ yet emotional problems in early years were not taken into account in these studies as a potential confounder. Analyses that consider the effects of both conduct and emotional problems in adolescence as confounders are therefore needed when examining associations between self-control in adolescence and mental health outcomes in adulthood.

Moreover, these longitudinal studies, so far, have not considered psychotic experiences as a mental health outcome. Self-control is thought to have developmental roots in a neural system subserving executive attention, the pivotal brain regions of which are focused on the orbitofrontal cortex, the anterior cingulate, and the paralimbic.^14–16^ Structural abnormalities in these regions involving deficits in attention, executive functioning, and behavior regulation^17–21^ have been reported in individuals with schizophrenia and first-episode psychosis.^19,22,23^ This overlap of neurobiological bases between self-control and psychotic phenomena suggests that there might be possible association and common underlying mechanisms between them. This has potential implications for intervention, because a focus on developing behavioral regulation in children is likely to be more effective for a range of outcomes than a narrow focus on schooling.^24^

Psychotic-like experiences (PLEs), which are attenuated hallucinatory and delusional symptoms, are experienced not only by patients with psychotic disorders but also by patients with nonpsychotic disorders and by a substantial part of general population.^25^ Population-based studies have shown that PLEs are quite common in the community through childhood and adolescence^26^ to adulthood^25^ and midlife.^27^ A recent meta-analysis reported that around 8% of the general adult population experienced psychotic phenomena.^25^ PLEs in the general population are generally transient and only a small proportion (8–10%) evolve into psychotic and nonpsychotic disorders each year,^28^ but they may share an etiological background with psychotic disorders.^25,29–31^ The PLEs phenotype is not a clinical outcome but has the important advantage, from a research perspective, of (1) being much more prevalent than schizophrenia and (2) not being dependent on help-seeking, thus allowing for true population-based epidemiological enquiry. These advantages mean that PLEs are suitable for epidemiological research into the pathophysiology, course, and treatment of psychotic symptoms in representative population-based contexts.^32^

The Medical Research Council National Survey of Health and Development (NSHD, the 1946 British birth cohort study) is one of the longest continuously running studies of human development and aging in the world^33^ and offers an excellent opportunity to test associations between adolescent self-control and future mental health outcomes. In this study, school teachers rated the behavior of participants when they were aged 13 and 15 years, based on 22 different questions. In a recent study,^34^ self-control was identified from these ratings as a separate dimension from conduct and emotional problems, and poor self-control in adolescence was found to be significantly associated with lower midlife memory. In the present study, we investigated associations between adolescent self-control and affective symptoms and psychotic-like symptoms at age 53 years. We hypothesized that adolescent self-control would be inversely associated with symptoms of anxiety and depression and psychotic-like symptoms in midlife.

## Methods

The NSHD is a prospective cohort study, originally consisting of 5362 individuals born in England, Scotland, and Wales in 1 week in March 1946. The cohort was stratified for social class such that all births to the wives of agricultural and nonmanual workers in that week were included, along with 1 in 4 births to the wives of manual workers, the majority occupational group in postwar Britain. This cohort has been prospectively studied 23 times, most recently at ages 60–64 years.^35^ At age 53 years, 3035 study members were successfully contacted. Major reasons for nonresponse included death (*n* = 469), temporary or permanent refusal (*n* = 948), participants who were temporarily or permanently abroad (*n* = 580), and participants who could not be traced (*n* = 330).^36^ Ethical approval for this study was granted by the North Thames Multicentre Research Ethics Committee, and all participants provided written informed consent.

### Midlife Symptom Assessment

Symptoms of anxiety and depression were self-rated at age 53 years using the 28-item version of the General Health Questionnaire (GHQ-28).^37^ Study members rated their experience over the previous few weeks (eg, “Have you recently lost much sleep over worry?”). Each individual item was scored using a 1–4 point Likert scale, and a total score was calculated (range 29–109; mean: 45.3, SD = 9.6). To test whether any association was confined to severe symptoms, we also repeated the analysis with the GHQ-28 Severe Depression (D) subscale only, which contains items about worthlessness, hopelessness, and suicidality.^37^ Of those contacted at age 53 (*n* = 3035), 2902 participants successfully completed the GHQ-28. Study members without GHQ-28 data at age 53 years for any reason were more likely to be male, lower social class assessed by occupational group of father in childhood, to have lower cognitive ability at age 8 years, and to score lower on self-control, and higher on conduct and emotional problems in adolescence (all *P* < .001).

At age 53 years, study members also rated possible PLEs over the previous 12 months using 5 screening-level items from the Psychosis Screening Questionnaire (PSQ).^38^ These questions have been used in previous studies of the British adult population to establish the prevalence of broadly defined PLEs.^39^ As in previous surveys, more than half of respondents gave a positive response to the hypomania item “Have there been times when you felt very happy without a break for days on end?”). Further, 643 respondents (22.0%) rated themselves as “unsure” on this item. As a result of this apparent lack of specificity, this item was eliminated from this analysis. Responses to the 4 remaining PSQ items referring to symptoms of thought interference (Have you felt that your thoughts were directly interfered with or controlled by some outside force or person?), persecution (Have there been times when you felt people were against you?), strange experiences (Have there been times when you felt that something strange was going on?), and hallucinations (Have there been times when you heard or saw things that other people couldn’t?) were coded as positive if the study member definitely reported that item and negative if the symptom was absent or was rated as unsure. Of those contacted at age 53 (*n* = 3035), 2918 participants successfully completed PSQ questions. As for those without GHQ-28 data, study members without PSQ data at age 53 years for any reason were more likely to be male, lower social class assessed by occupational group of father in childhood, to have lower cognitive ability at age 8 years, and to score lower on self-control and higher on conduct and emotional problems in adolescence (all *P* < .001).

### Adolescent Self-control, Conduct, and Emotional Problems

Teachers were asked to rate the behaviors of participants on a 3-category response scale where they had to compare the participants’ behaviors to those of “a normal child” at age 13 and 15 years using items that were forerunners of those used in the Rutter A scale.^40,41^ These data were subjected to separate exploratory factor analysis at these ages^34^ using item response theory methods in the statistical software package Mplus 6.1.^42^ Data were modeled as ordinal variables using weighted least squares means and variance adjusted estimator through probit link. Examination of Scree plots, eigenvalues, and model fit indices suggested a 3-factor solution (see table 1 in the Xu et al paper^34^ for descriptions of factor loadings and item wordings). Specifically, a factor of “self-control” was identified as a dimension separate from those of “conduct” (eg, disobedience, evading truth to keep out of trouble) and “emotional” (eg, gloomy and sad, extremely fearful) problems. The “self-control” factor, which correlates more with the conduct factor than the emotional factor, was defined by items relating to attitude to work, concentration, neatness in work, and not daydreaming in class.^34^ Factor scores were exported for each factor at age 13 and 15 years and summed to create overall scores for these dimensions, with a higher score indicating lower self-control and worse emotional and conduct problems. To facilitate interpretation, the new combined scales were then standardized to form *z* scores.

**Table 1.** Associations Between GHQ-28 Aged 53 y and Scores of Self-control, Conduct, and Emotional Problems Aged 13+15 y

	GHQ-28 Age 53											
Scores Derived From Teacher Rating	Unadjusted Association				Adjusted Association^a^				Adjusted Association^b^			
Latent Factors	Regression Coefficient	95% CI		*p* value	Regression Coefficient	95% CI		*p* value	Regression Coefficient	95% CI		*p* value
Male												
Self-control problem 13 + 15	0.108	−0.378	0.593	.664	−0.393	−1.144	0.357	.304	−0.414	−1.117	0.290	.249
Conduct problem 13 + 15	0.080	−0.424	0.585	.755	0.438	−0.273	1.149	.227	0.398	−0.270	1.065	.243
Emotional problem 13 + 15	0.534	0.020	1.047	.042	0.734	0.114	1.354	.020	0.772	0.192	1.353	.009
Female												
Self-control problem 13 + 15	1.058	0.439	1.678	.001	0.535	−0.379	1.448	.251	0.411	−0.431	1.253	.339
Conduct problem 13 + 15	0.622	0.038	1.207	.037	0.283	−0.522	1.088	.490	0.129	−0.612	0.871	.733
Emotional problem 13 + 15	0.711	0.137	1.284	.015	0.404	−0.285	1.094	.250	0.338	−0.299	0.975	.298

*Note*: ^a^Regression coefficient adjusted for each mutually adjusted score derived from teacher ratings at age 13 + 15 y, social class in childhood, and childhood cognitive function at age 8.

^b^Regression coefficient adjusted for each mutually adjusted score derived from teacher ratings at age 13 + 15 y, social class in childhood, childhood cognitive function at age 8, and the sum of PSQ symptoms.

### Childhood Cognitive Assessment

Childhood cognitive ability predicts midlife affective symptoms in NSHD,^43^ although this was explained by psychotic-like symptoms.^27^ Childhood cognitive ability at age 8 years was represented as the sum of 4 tests of verbal and nonverbal ability devised by the National Foundation for Education Research.^38^ These tests were (1) reading comprehension (selecting appropriate words to complete 35 sentences); (2) word reading (ability to read and pronounce 50 words); (3) vocabulary (ability to explain the meaning of 50 words); and (4) picture intelligence, consisting of a 60-item nonverbal reasoning test. We used confirmatory factor analysis to construct a scale summarizing these data. Model fit indices were chi-square = 63.145 with 1 degree of freedom, RMSEA = 0.121, CFI = 0.994, and TLI = 0.966. Factor scores were computed and then standardized to a mean of 0 with a SD of 1.

### Early-Life Socioeconomic Factors

Early-life socioeconomic factors, as assessed by occupational social class of the father when study members were aged 11 years, or if this was unknown, at 4 or 15 years, were considered possible confounders for any association between adolescent self-control, conduct, and emotional problems and adult psychopathologic symptoms.

### Statistical Analysis

Associations between total score for each of adolescent self-control, conduct, and emotional problems and total GHQ-28 score and the GHQ-28 Severe Depression subscale score were tested using multivariable regression models. Because the self-control × sex interaction terms for GHQ-28 total score and GHQ-28 Severe Depression subscale score were significant (*P* = .018, *P* = .006, respectively), these models were stratified by sex. The first model tested unadjusted associations between these variables. The second model adjusted for father’s social class and childhood cognitive ability. In the third model, we additionally adjusted for total number of PSQ symptoms, because they are associated with GHQ score in NSHD.^27^

We then used logistic regression to test associations between adolescent self-control, conduct, and emotional problems and each PSQ symptom (thought interference, persecution, strange experiences, and hallucinations), using those without these symptoms as reference groups. We then adjusted for sex (self-control × sex interaction terms for each PSQ symptom: thought interference, *P* = .276; persecution, *P* = .377; strange experiences, *P* = .361; hallucination, *P* = .083), father’s social class and childhood cognitive ability (as in the model for the GHQ-28 outcome), and additionally the GHQ-28 score. All models were estimated using IBM SPSS version 21.0 for Windows (New York).

## Results

The GHQ-28 at age 53 was completed by 1423 men (mean score [SD]: 43.7 [8.6]) and 1479 women (mean score [SD]: 46.9 [10.3]), *P* < .001. Corresponding values for manual vs nonmanual social class of origin were 45.3 (9.7) and 45.4 (9.6), *P* = .75. The PSQ at age 53 was completed by 1430 men and 1488 women. The 4 PLEs were positively endorsed with the following prevalence: thought interference, *n* = 302 (10.3%); persecution, *n* = 397 (13.6%); strange experiences, *n* = 216 (7.4%); hallucinations, *n* = 107 (3.7%). Less than 1% of study members endorsed all 4 symptoms. Symptom frequencies were equal in men and women (χ^2^ = 2.94, *d* *f* = 4, *P* = .57), and endorsement of one or more symptoms was unrelated to manual vs nonmanual social class of origin (χ^2^ = 0.87, *d* *f* = 1, *P* = .35).

### Adolescent Self-control and Midlife Symptoms of Anxiety/Depression

When unadjusted associations were examined using multivariable regression, adolescent self-control problems were associated at 5% significance with higher total GHQ-28 score in females (regression coefficient = 1.06, 95% CI = 0.44–1.68, *P* = .001); this was also the case for conduct problems (regression coefficient = 0.62, 95% CI = 0.04–1.21, *P* = .037). After mutually adjusting the adolescent teacher ratings, and additionally adjusting for social class in childhood, childhood cognitive ability, and the total number of PSQ symptoms at age 53 years, adolescent self-control problems in females did not remain significantly associated with symptoms of anxiety and depression at age 53 years (regression coefficient = 0.41, 95% CI = −0.43 to 1.25, *P* = .339) (table 1).

### Adolescent Self-control and Midlife Severe Depression

When unadjusted associations were examined using multivariable regression, adolescent self-control problems were associated at 5% significance with severe depression in females only (regression coefficient = 0.33, 95% CI = 0.17–0.50, *P* < .001). After mutually adjusting the adolescent teacher ratings, and additionally adjusting for social class in childhood, childhood cognitive ability, and the total number of PSQ symptoms at age 53 years, adolescent self-control problems in female did not remain significantly associated with severe depression at age 53 years (regression coefficient = 0.09, 95% CI = −0.14 to 0.31, *P* = .434) (table 2). This was also the case when the self-control score was split into tertiles, and the highest tertile compared to the lowest on the GHQ-28 (*P* = .148).

**Table 2.** Associations Between GHQ-28 Severe Depression (D) Subscale Aged 53 y and Scores of Self-control, Conduct, and Emotional Problems Aged 13+15 y

Scores Derived From Teacher Rating	GHQ-28-D-Scale Age 53											
	Unadjusted Association				Adjusted Association^a^				Adjusted Association^b^			
Latent Factors	Regression Coefficient	95% CI		*p* value	Regression Coefficient	95% CI		*p* value	Regression Coefficient	95% CI		*p* value
Male												
Self-control problem 13 + 15	0.039	−0.092	0.169	.559	−0.136	−0.340	0.067	.189	−0.141	−0.333	0.051	.151
Conduct problem 13 + 15	0.057	−0.079	0.193	.409	0.140	−0.053	0.332	.155	0.127	−0.055	0.309	.173
Emotional problem 13 + 15	0.100	−0.038	0.238	.156	0.153	−0.014	0.321	.073	0.163	0.005	0.321	.044
Female												
Self-control problem 13 + 15	0.334	0.169	0.498	<.001	0.110	−0.133	0.353	.375	0.089	−0.135	0.313	.434
Conduct problem 13 + 15	0.191	0.036	0.345	.016	0.113	−0.101	0.326	.301	0.058	−0.139	0.255	.564
Emotional problem 13 + 15	0.227	0.075	0.379	.003	0.150	−0.033	0.333	.107	0.120	−0.049	0.289	.165

Note^a^Regression coefficient adjusted for each mutually adjusted score derived from teacher ratings at age 13 + 15 y, social class in childhood, and childhood cognitive function at age 8.

^b^Regression coefficient adjusted for each mutually adjusted score derived from teacher ratings at age 13 + 15 y, social class in childhood, childhood cognitive function at age 8, and the sum of PSQ symptoms.

### Adolescent Self-control and PLEs in Midlife

The 4 PLEs had the following prevalence: thought interference, *n* = 302 (10.3%); persecution, *n* = 397 (13.6%); strange experiences, *n* = 216 (7.4%); and hallucinations, *n* = 107 (3.7%). Less than 1% of participants experienced all 4 symptoms. A significant unadjusted association was found only between adolescent self-control problems and midlife hallucinations (OR = 1.54, 95% CI = 1.24–1.91, *P* < .001) (table 3). The strength of this association was unchanged after adjusting for sex, social class in childhood, childhood cognitive ability, adolescent conduct and emotional problems, and GHQ-28 score (OR = 1.55, 95% CI = 1.09–2.18, *P* = .013) (table 3).

**Table 3.** Associations Between Each Psychotic Experience Aged 53 y and Scores of Self-control, Conduct, and Emotional Problems Aged 13+15 y

Scores Derived From Teacher Rating	Thought Interference			Persecution			Strange Experience			Hallucination		
	*n* = 302 (10.3%)			*n* = 397 (13.6%)			*n* = 216 (7.4 %)			*n* = 107 (3.7 %)		
Latent Factors	OR	95% CI	*p* value	OR	95% CI	*p* value	OR	95% CI	*p* value	OR	95% CI	*p* value
Unadjusted associations												
Self-control problem 13 + 15	1.08	0.94–1.23	.293	1.12	0.99–1.26	.068	1.17	1.00–1.38	.058	1.54	1.24–1.91	<.001
Conduct problem 13 + 15	1.07	0.94–1.22	.316	1.12	1.00–1.26	.061	1.15	0.98–1.34	.090	1.22	1.00–1.50	.052
Emotional problem 13 + 15	0.96	0.84–1.10	.594	1.05	0.93–1.18	.412	1.03	0.88–1.21	.729	1.08	0.87–1.33	.490
Adjusted associations^a^												
Self-control problem 13 + 15	1.02	0.83–1.26	.839	0.99	0.82–1.18	.884	1.02	0.80–1.31	.859	1.53	1.09 – 2.15	.014
Conduct problem 13 + 15	1.01	0.83–1.21	.950	1.11	0.94–1.30	.212	1.07	0.85–1.33	.580	0.91	0.67–1.23	.524
Emotional problem 13 + 15	0.93	0.79–1.10	.385	1.05	0.91–1.21	.533	1.01	0.83–1.23	.901	0.91	0.70–1.19	.495
Adjusted associations^b^												
Self-control problem 13 + 15	1.03	0.82–1.27	.818	0.98	0.81–1.19	.822	1.04	0.80–1.36	.758	1.55	1.09–2.18	.013
Conduct problem 13 + 15	0.98	0.80–1.19	.803	1.07	0.90–1.28	.434	1.02	0.81–1.30	.847	0.90	0.66–1.22	.491
Emotional problem 13 + 15	0.88	0.74–1.05	.160	1.00	0.86–1.16	.967	0.95	0.77–1.17	.615	0.90	0.69–1.18	.443

Note:^a^OR adjusted for each mutually adjusted score derived from teacher ratings at age 13 + 15 years, sex, social class in childhood, and childhood cognitive function at age 8.

^b^OR adjusted for each mutually adjusted score derived from teacher ratings at age 13 + 15 years, sex, social class in childhood, childhood cognitive function at age 8, and GHQ-28 total scores at age 53.

## Discussion

Using data from a national birth cohort, we showed that self-control problems in adolescence predicted hallucinatory experiences in midlife after controlling for the effects of adolescent conduct and emotional problems, symptoms of anxiety and depression in midlife, sex, social class of origin, and childhood cognition. We did not find any significant independent association between adolescent self-control and midlife symptoms of anxiety and depression, even when severe symptoms only were used as an outcome. The prevalence of PLEs in this study was 22.3%, which was higher than that reported by a recent meta-analysis,^25^ but similar to the results by studies using same instrument.^39,44^ In particular, the British National Morbidity Survey, which was nationwide adult population survey, reported similar prevalence of each PSQ items (thought insertion: 9.0%; strange experiences: 8.9%; hallucinations: 4.2%), except for the higher prevalence of persecution (21.2%),^39^ to those in our current study (thought interference *n*: 10.3%; persecution: 13.6%; strange experiences: 7.4%; hallucinations: 3.7%).

Strengths of our study include the use of a national population-based sample and independently and prospectively rated adolescent mental health, as well as availability of a range of potential confounders. Limitations are that our main analyses stemmed from self-reported responses from a small number of broad questions about PLEs. It is possible that individuals with lower cognitive scores might be more likely to misinterpret or misread questions about such experiences in self-reported questionnaires. However, this explanation seems unlikely given that childhood cognition, which correlates with adult,^45^ midlife^46^ and later life^47^ cognition, was controlled in the present study. Another limitation is that the information about illicit drug use in adolescence and adulthood, which might be considered as a confounder,^3^ was not available in the present study.

A further limitation of the present study is that individuals may have had PLEs at times outside of the PSQ assessment window. In the NSHD, the PSQ was only administered at age 53 years; prior to that only clinical diagnoses^48^ or more narrowly defined clinical symptoms^27^ were elicited. Thus, the no-symptom group may contain some false-negative findings, which would lead to a conservative bias in the effects. Conversely, as these experiences are more common during adolescence and early adulthood than in later years,^26,49^ individuals who report them in late adulthood may represent a “poor prognosis” subset whose experiences did not dissipate with age, inflating the association between adolescent self-control and late-life experiences. Sample attrition would be expected to lead to underrepresentation both of individuals with poorer self-control and of individuals with psychotic symptoms, potentially producing a conservative bias. Wadsworth et al conducted an in-depth analysis of factors that predicted nonresponse at age 53 years; these included male gender, low cognitive test score at age 8 years, behavior at age 15 years, and educational, stress, and social factors during early adulthood.^36^ It is likely that some of these factors may also be related to the likelihood of experiencing psychotic-like symptoms. However, while this may have reduced statistical power, we have no reason to believe that this would have altered the pattern of associations observed here.

Recent neuroimaging studies have investigated the neurobiological basis of self-control. A brain morphological study which examined a large sample of healthy early adolescents revealed that higher self-control was significantly associated with larger volume of the orbitofrontal cortex and hippocampus.^50^ A number of neuroimaging studies have reported structural and functional abnormalities in the orbitofrontal cortex and hippocampus in patients with schizophrenia and first-episode of psychosis.^22,23,51^ Furthermore, some longitudinal studies in individuals at high risk of developing psychosis have demonstrated progressive orbitofrontal cortex volume decrease during the transition from subclinical psychotic symptoms to clinical psychosis.^52,53^ The orbitofrontal cortex is thought to modulate human behavior through a stimulus-reinforcer association learning process, and it is also involved in various cognitive functions such as executive functioning and decision making,^54–57^ these deficits have been observed in individuals with psychosis and subclinical psychotic symptoms.^58–60^

Associations between adolescent self-control and midlife psychotic-like symptoms only reached conventional levels of significance for hallucinations, although this was not a matter of power since hallucinations showed the lowest frequency of these PSQ items; and it is also worth noting that associations for *all* psychotic-like symptoms were in the positive direction. Our current understanding of the pathophysiology of schizophrenia posits that developmental differences reflect aberrance in early brain development, the full effects of which do not become apparent until late adolescence or adulthood.^61^ The presence of early low self-control among individuals who will later experience hallucinatory-like experiences suggest that this model can be applied more broadly throughout the spectrum of risk for psychosis.

It is worth commenting on the lack of change in the association between self-control and hallucinations when this was adjusted for childhood cognition, because the latter is frequently associated with adult psychotic experiences. It is possible that the executive component of cognition that is thought to explain this association was better accounted for by the self-control variable.

## Conclusions

Lower self-control in adolescence may be a risk factor for hallucinatory experiences in adulthood.

## Funding

Funding for the National Survey of Health and Development is provided by the Medical Research Council. This work was supported by the Medical Research Council (to M.R.), Wellcome Trust grant (088869/Z/09/Z), the Ministry of Education, Culture, Sports, Science and Technology of Japan Scientific Research Grant on Innovative Area (to A.N., 23118004; Adolescent Mind & Self-Regulation), and the Japanese Society for the Promotion of Scientific Research Grant (to A.N., Strategic Young Researcher Overseas Visits Program for Accelerating Brain Circulation).

## Declaration of Interests

None declared.
